# Screening for Selective Anticancer Activity of 65 Extracts of Plants Collected in Western Andalusia, Spain

**DOI:** 10.3390/plants10102193

**Published:** 2021-10-15

**Authors:** José Manuel Calderón-Montaño, Sara María Martínez-Sánchez, Víctor Jiménez-González, Estefanía Burgos-Morón, Emilio Guillén-Mancina, Julio José Jiménez-Alonso, Patricia Díaz-Ortega, Felipe García, Abelardo Aparicio, Miguel López-Lázaro

**Affiliations:** 1 Department of Pharmacology, Faculty of Pharmacy, University of Seville, 41012 Seville, Spain; vjimenez3@us.es (V.J.-G.); eburgos1@us.es (E.B.-M.); eguillen@us.es (E.G.-M.); juljimalo@alum.us.es (J.J.J.-A.); patdiaort@alum.us.es (P.D.-O.); 2 Department of Vegetal Biology and Ecology, Faculty of Pharmacy, University of Seville, 41012 Seville, Spain; smmartinez2@ucam.edu (S.M.M.-S.); fgm@us.es (F.G.); abelardo@us.es (A.A.); 3 Department of Food Technology and Nutrition, San Antonio Catholic University, 30107 Murcia, Spain

**Keywords:** cancer, lung cancer, selectivity, *Tetraclinis articulata* (Vahl) Mast., Cupressaceae

## Abstract

Finding cytotoxic drugs with a high selectivity towards cancer cells is crucial to improve the low survival rates of patients diagnosed with metastatic cancers. Since plants are an important source of anticancer drugs, we have screened 65 extracts from 45 plants collected in several areas of Western Andalusia (Spain) for cytotoxic activity on lung cancer cells versus lung normal cells. An extract from the leaves of *Tetraclinis articulata* (Vahl) Mast. (Cupressaceae) showed a marked cytotoxicity (IC50 = 0.37 ± 0.03 μg/mL) and selectivity (selectivity index = 378.3) against the lung cancer cells; cisplatin, 5-fluorouracil, and an extract from the leaves of *Taxus baccata* L. (Taxaceae) were less cytotoxic and selective. Extracts from *Cascabela thevetia* (L.) Lippold (Apocynaceae), *Frangula alnus* Mill. (Rhamnaceae), *Iberis ciliata* subsp. *contracta* (Pers.) Moreno (Brassicaceae), *Juniperus macrocarpa* Sm (Cupressaceae), and *Pancratium maritimum* L. (Amaryllidaceae) also showed selective cytotoxicity (selectivity index > 10). Active extracts were also tested against a panel of cancer cell lines from a variety tissues. The plants identified in this work are potential sources of natural compounds with selective toxicity towards cancer cells.

## 1. Introduction

Despite the recent approval of new anticancer treatments, metastasis continues to be an incurable disease for most cancer patients. The limited efficacy of the existing therapies is reflected in the poor survival rates of patients diagnosed with the most common metastatic cancers [1]. For example, distant metastases occur in 57% of patients diagnosed with lung cancer, and only 6% of them survive 5 years after diagnosis [1]. The percentages are also low for other common metastatic cancers. The five-year relative survival rates for patients with distant metastases are 30% in prostate cancer, 28% in breast cancer, 14% in colorectal cancer, 27% in melanoma, 13% in renal cancer, 30% in ovarian cancer, 17% in cancers of the uterine corpus, 17% in cancers of the uterine cervix, 6% in bladder cancer, 5% in esophageal cancer, 3% in liver cancer, and 3% in pancreatic cancer [1]. Many patients with metastases do not overcome the disease despite surviving five years after diagnosis.

Understanding why the current treatments rarely cure patients with disseminated disease is important to discover better therapies. When one treats cancer cells with specific concentrations of the available anticancer drugs and examines the cells under the microscope, one generally observes a massacre. All cancer cells die in response to most treatments. However, these drugs also kill normal cells at similar concentrations. The consequence of this limited selectivity is that patients cannot receive the drug doses needed to kill all their cancer cells; such doses would also kill their normal body cells and would be lethal. Although oncology patients generally receive the maximum tolerated doses, these doses are usually insufficient to reach the drug concentrations required to eradicate their cancer cells. The surviving cancer cells continue to proliferate and eventually lead to a fatal outcome. Finding drugs with a high selectivity towards cancer cells is crucial to develop more effective treatments for patients with metastasis [2,3,4].

Several plants have provided useful drugs for the treatment of a variety of cancers, including lung cancer [5,6,7]. For example, the diterpene paclitaxel (isolated from the bark of *Taxus brevifolia* Nutt., Taxaceae) and its semisynthetic derivative docetaxel are FDA-approved drugs for the treatment of non-small-cell lung cancer [8,9]. Vinorelbine, a semisynthetic analog of the vinca alkaloids (isolated from *Catharanthus roseus* G. Don., Apocynaceae), is also approved for patients with this type of cancer [9]. Etoposide (a semi-synthetic analogue of the natural lignan podophyllotoxin, isolated from *Podophyllum* species, Podophyllaceae) and topotecan (an analogue of the quinoline alkaloid camptotethin, isolated from *Camptotheca acuminata* Decne, Nyssaceae) are approved for patients with small cell lung cancer. Due to the fact that several plants have provided useful anticancer agents, we recently used lung cancer cells and lung normal cells to evaluate the selective anticancer activity of 57 extracts from plants collected in Grazalema Natural Park (Andalusia, Spain) [10]. Using a similar experimental approach, we have evaluated the selective anticancer activity of 65 extracts from 45 new plants collected in several areas of Western Andalusia, and report the results in this article.

## 2. Results and Discussion

Due to the fact that patients with metastatic cancers need selective anticancer drugs, we have searched for potential sources of selective anticancer drugs in 45 plants growing in Western Andalusia, Spain. After collecting the plant material and preparing 65 extracts, we used lung cancer cells (A549) and lung normal cells (MRC-5) to evaluate their selective cytotoxicity with the MTT assay. A549 cells are epithelial cells and MRC-5 cells are fibroblastic cells. However, since normal epithelial cells are difficult to maintain and expand in culture, we selected this widely used normal cell line for our screening [11,12,13].

**Figure 1:**
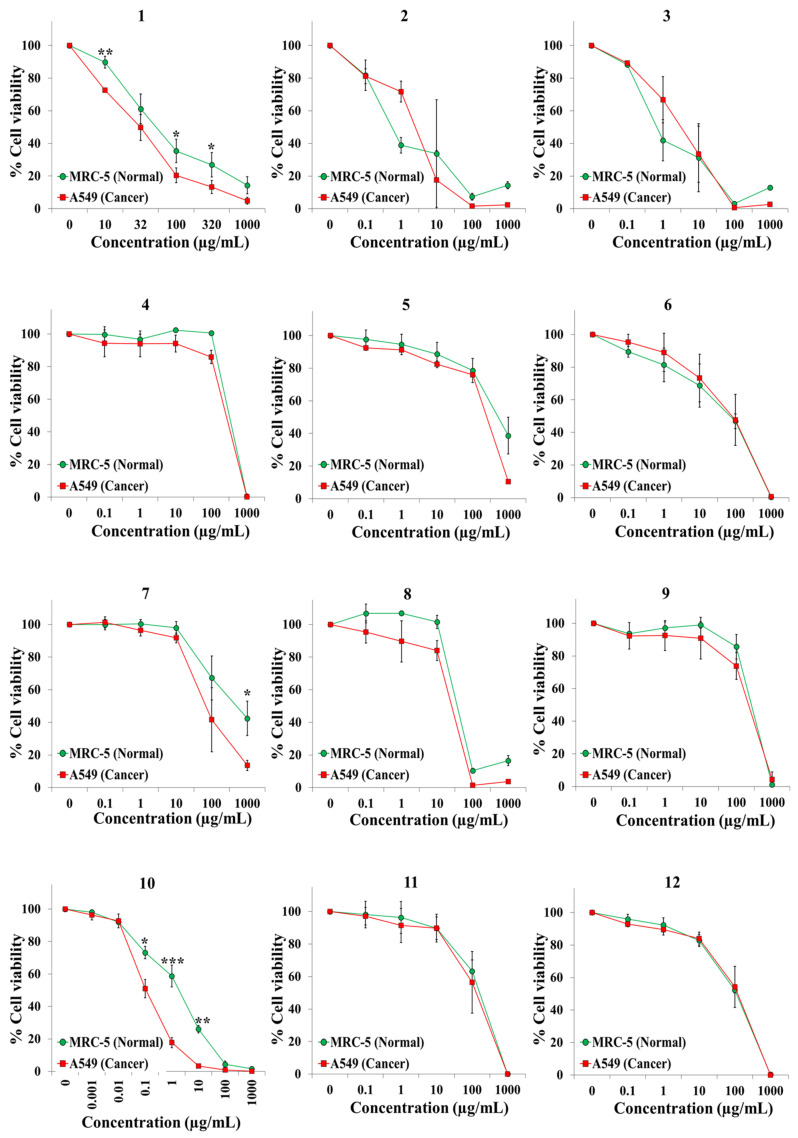
Screening for selective cytotoxic activity of plant extracts **1**–**12** on A549 lung cancer cells and MRC-5 lung normal cells. The cells were exposed for 72 h to the extracts and cell viability was determined with the MTT assay.

**Figure 2:**
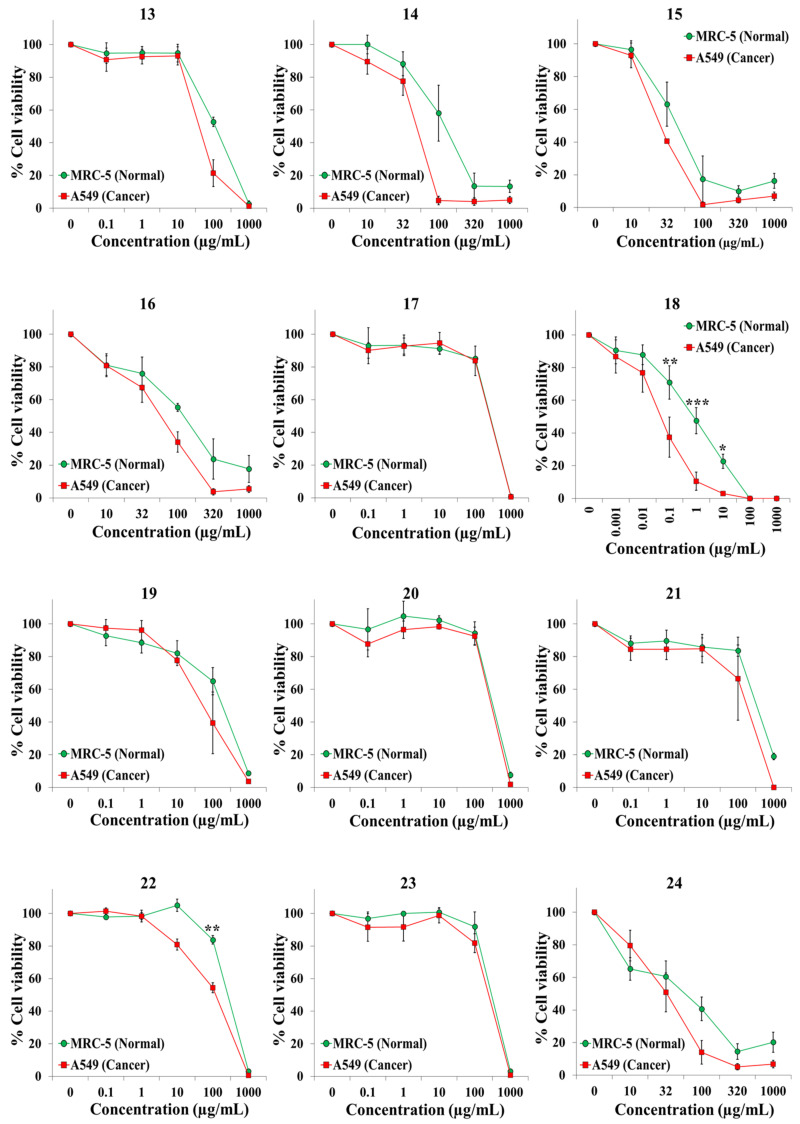
Screening for selective cytotoxic activity of plant extracts **13**–**24** on A549 lung cancer cells and MRC-5 lung normal cells. The cells were exposed for 72 h to the extracts and cell viability was determined with the MTT assay.

**Figure 3:**
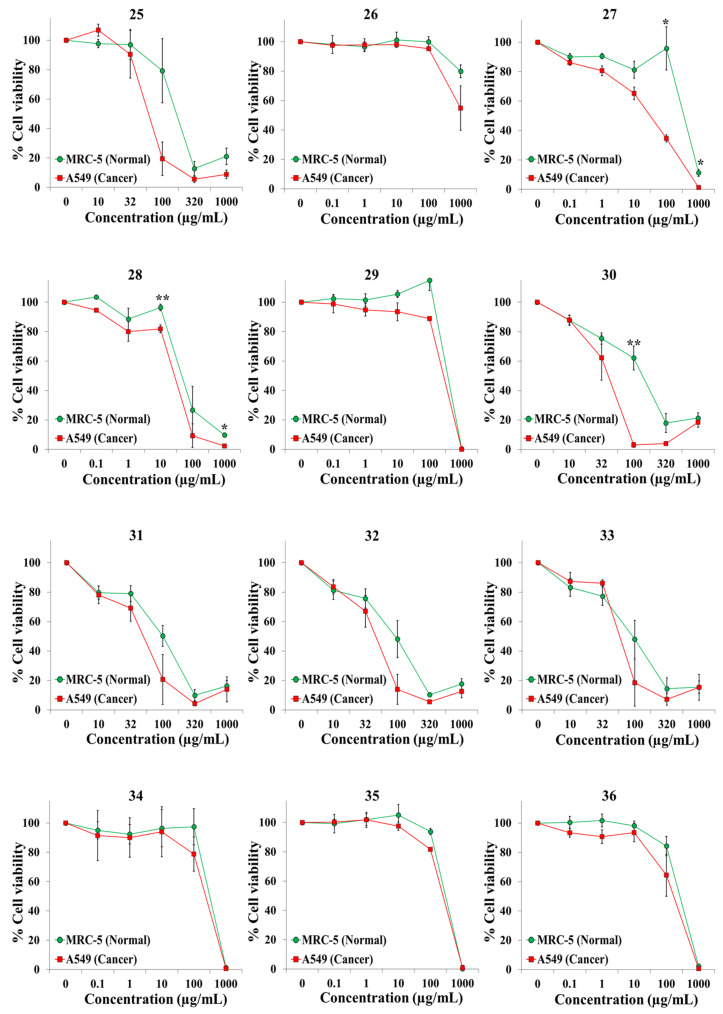
Screening for selective cytotoxic activity of plant extracts **25**–**36** on A549 lung cancer cells and MRC-5 lung normal cells. The cells were exposed for 72 h to the extracts and cell viability was determined with the MTT assay.

**Figure 4:**
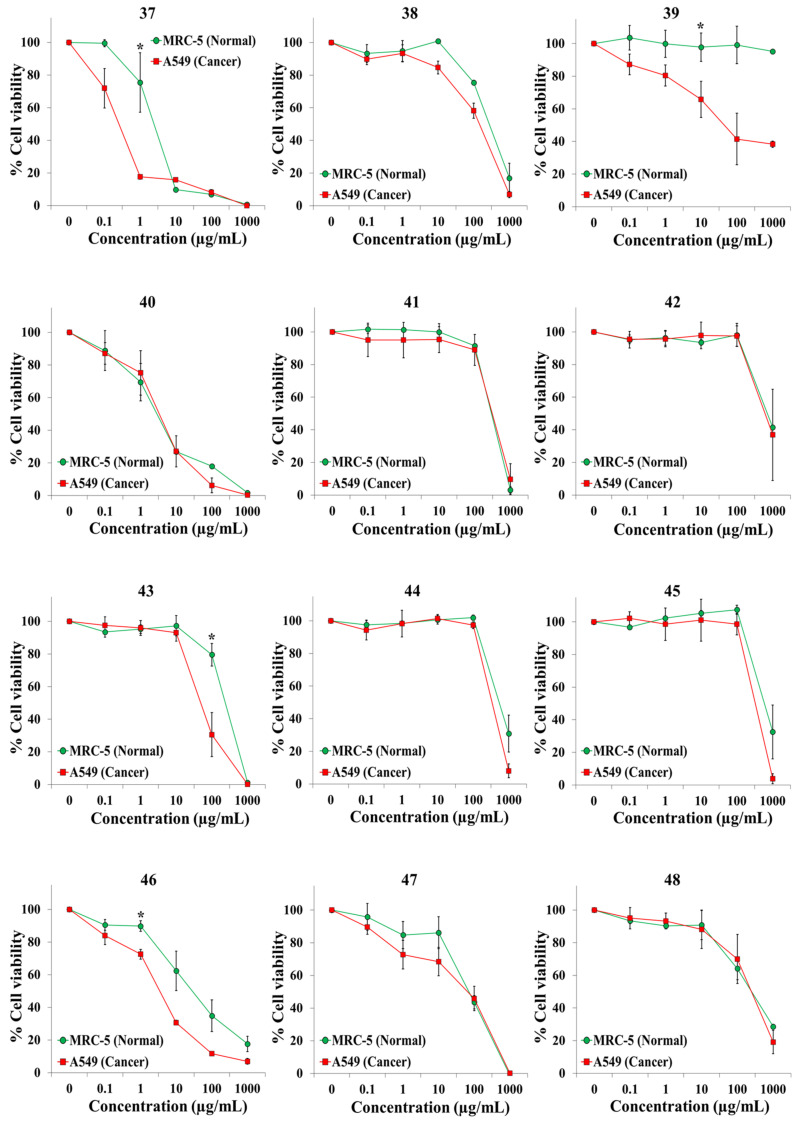
Screening for selective cytotoxic activity of plant extracts **37**–**48** on A549 lung cancer cells and MRC-5 lung normal cells. The cells were exposed for 72 h to the extracts and cell viability was determined with the MTT assay.

**Figure 5:**
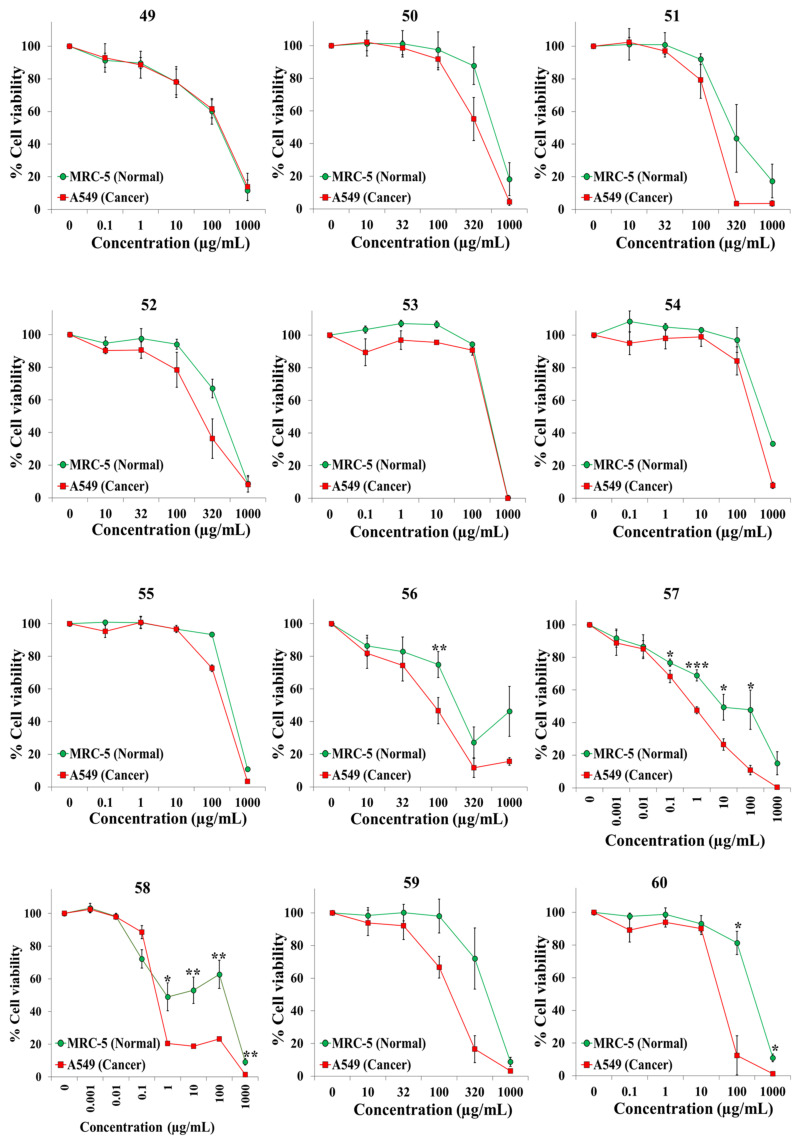
Screening for selective cytotoxic activity of plant extracts **49**–**60** on A549 lung cancer cells and MRC-5 lung normal cells. The cells were exposed for 72 h to the extracts and cell viability was determined with the MTT assay.

**Figure 6:**
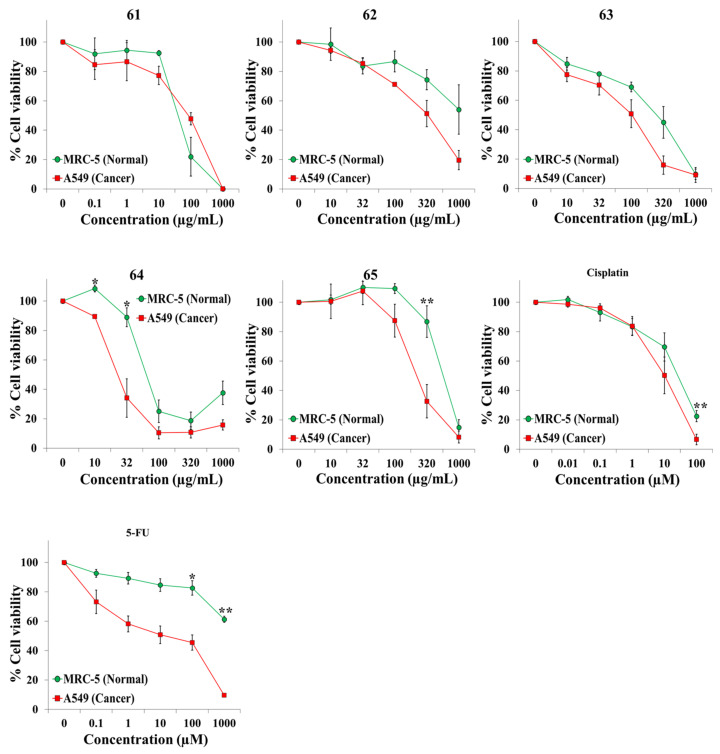
Screening for selective cytotoxic activity of plant extracts **61**–**65**, cisplatin, and 5-fluorouracil (5-FU) on A549 lung cancer cells and MRC-5 lung normal cells. The cells were exposed for 72 h to the extracts or anticancer drugs and cell viability was determined with the MTT assay.

We exposed both cell lines for 72 h to at least five concentrations of the extracts and the anticancer drugs cisplatin and 5-fluorouracil (5-FU). Table 1 shows the botanical names in alphabetical order, the plant families and other pertinent information on the 45 plant species. It also shows an identification number for each extract, the IC50 value for each cell line, and the selectivity index. Dose–response curves for the 65 extracts and positive controls are provided in Figure 1, Figure 2, Figure 3, Figure 4, Figure 5 and Figure 6; these curves allow for the visualization of their cytotoxic profile over a wide concentration range.

Results show that several extracts induced selective cytotoxicity towards the cancer cell line, including the extract from the leaves of *Cascabela thevetia* (L.) Lippold (Apocynaceae) (**10**), the extract from the leaves of *Digitalis purpurea* L. (Plantaginaceae) (**18**), the extract from the bark of *Frangula alnus* Mill. (Rhamnaceae) (**27**), the extract from the whole plant *Iberis ciliata subsp. contracta* (Pers.) Moreno (Brassicaceae) (**37**), the extract from the seeds of *Juniperus macrocarpa* Sm. (Cupressaceae) (**39**), the extract from the bulb of *Pancratium maritimum* L. (Amaryllidaceae) (**46**), and the extract from the leaves of *Tetraclinis articulata* (Vahl) Mast. (Cupressaceae) (**58**). The anticancer drugs cisplatin and 5-fluorouracil, and an extract from the leaves of *Taxus baccata* L. (Taxaceae) (**57**) also showed selectivity against the cancer cells (Table 1). The extract from the leaves of *Tetraclinis articulata* (Vahl) Mast. (**58**) (Figure 5) showed the most relevant activity; it was more cytotoxic against the lung cancer cells (IC50 = 0.37 ± 0.03 µg/mL) than against the lung normal cells (IC50 = 129.5 ± 64.0 µg/mL), displaying a selectivity index of 378.3 ± 178.1. No significant differences were observed when the cytotoxicity of this extract was tested with the MTT assay and the resazurin assay (Figure S1). Photographs of this plant and the plant material used to prepare the extract are shown in Figure S2. The extract from the leaves of *Taxus baccata* L. (Taxaceae) was also more cytotoxic against the lung cancer cells (IC50 = 0.86 ± 0.27 µg/mL) than against the lung normal cells (IC50 = 146.9 ± 87.8 µg/mL), displaying a selectivity index of 157.3 ± 110.6. Several extracts (e.g., **4**, **6**, **9**, **11**, **12**, **17**, **20**, **23**, **34**, **35**, **41**, **42**, **48**, **49**, **53**) were cytotoxic but were not selective against the A549 lung cancer cells.

**Figure 7:**
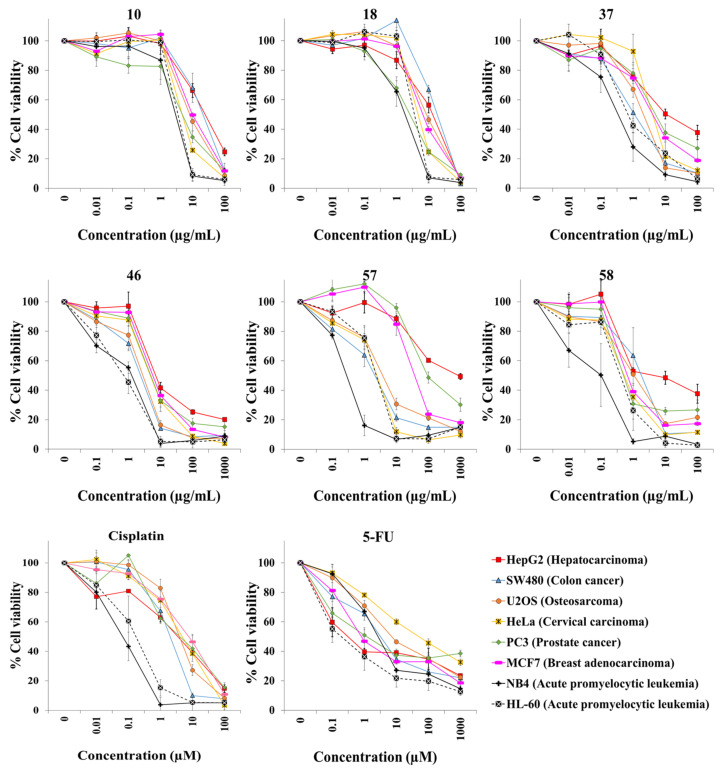
Cytotoxicity of selected plant extracts (**10**, **18**, **37**, **46**, **57**, and **58**), cisplatin, and 5-FU against six cancer cell lines derived from solid tumors and two acute promyelocytic leukemia cell lines. Cells were treated for 72 h, and cell viability was evaluated with the resazurin assay.

Extracts (**10**, **18**, **37**, **46**, **57**, and **58**), and the anticancer drugs cisplatin and 5-fluorouracil, were tested with the resazurin assay against eleven additional cell lines: two leukemia cell lines, six cancer cell lines derived from solid tumors of different tissues (liver, colon, bone, cervix, prostate, and breast), and three genetically modified skin cell lines with increasing degrees of malignancy (Table 2 and Figure 7). Due to the fact that the leukemia cell lines grow in suspension, we determined cell viability with the resazurin assay. Unlike the MTT assay, this assay does not require removal of the culture medium. The extract from the leaves of *Tetraclinis articulata* (Vahl) Mast. (**58**) displayed the highest cytotoxic activity, with IC50 values between 0.2 and 2.1 μg/mL against seven of the eight cancer cell lines. No clear differences in cytotoxicity were found for any extract or compound in the three skin cell lines with increasing degrees of malignancy (BJ-hTERT, BJ-SV40T, and BJ-RASV12) [14] (Table 2).

Due to the fact that *Tetraclinis articulata* (Vahl) Mast. extract (**58**) showed a high cytotoxicity against several cancer cell lines, we acquired additional cancer cell lines from a variety of tissues to further evaluate the activity of the extract. Fourteen human cancer cell lines were treated with several concentrations of **58** for 96 h and cell viability was estimated with the resazurin assay. Results show that this extract was cytotoxic against all the cancer cell lines, with IC50 values in the range 4.3–4.9 µg/mL (Table 3).

Several types of phytochemicals may be involved in the selective anticancer activity shown by the most active extracts. Cardiac glycosides may be responsible for the selective cytotoxicity shown by the extracts from the leaves of *Cascabela thevetia* (L.) Lippold (**10**) and the leaves of *Digitalis purpurea* L. (**18**). We, and others, have previously observed that plants with cardiac glycosides, and several cardiac glycosides (e.g., digitoxin), induce potent and selective cytotoxic effects against several types of cancer cells, including lung cancer cells [15,16,17,18,19,20,21,22,23]. Isoquinoline alkaloids may participate in the cytotoxicity and selectivity observed for the extract from the bulb of *Pancratium maritimum* L. (**46**) [24,25]. The cytotoxicity and selectivity of our extract from the leaves of *Taxus baccata* L. (**57**) were probably mediated by different taxane-type diterpenes, including paclitaxel [26,27]. Diterpenes (e.g., ferruginol and sandaracopimaric acid.), monoterpenes (e.g., limonene and carveol), and sesquiterpenes (e.g., β-caryophyllene and humulene) may participate in the cytotoxicity of the extract from the leaves of *Tetraclinis articulata* (Vahl) Mast. [28,29,30,31,32,33].

In summary, because patients with advanced cancers need selective anticancer drugs, we have searched for potential sources of selective anticancer drugs in a variety of plants. After collecting 45 plants and preparing 65 extracts, we used a cancer cell line and a normal cell line from the same tissue to detect selective cytotoxic activity [2,3,4]. Several extracts induced selective cytotoxicity towards the cancer cell line, including extracts from *Cascabela thevetia* (L.) Lippold (Apocynaceae), *Frangula alnus* Mill. (Rhamnaceae), *Iberis ciliata* subsp. *contracta* (Pers.) Moreno (Brassicaceae), *Juniperus macrocarpa* Sm (Cupressaceae), *Pancratium maritimum* L. (Amaryllidaceae), and *Tetraclinis articulata* (Vahl) Mast. (Cupressaceae). Due to the fact that the selective anticancer activity of *Tetraclinis articulata* (Vahl) Mast was high, we evaluated and observed cytotoxic activity in an additional 22 cancer cell lines, representing the most common types of cancer. Our study shows that several plants are promising sources for the isolation and the development of new anticancer drugs with cancer-selective toxicity.

## 3. Materials and Methods

### 3.1. Plant Material

All plants were collected by F. García between November 2012 and April 2013 in several areas of Sevilla, Cadiz, and Huelva (Andalusia, Spain). Collection was non-destructive and plant specimens (5–110 g) were carefully selected to avoid any damage that could affect the conservation of any species. A voucher specimen was deposited in the herbarium at the CITIUS II Celestino Mutis (Center for Research, Technology, and Innovation of the University of Seville). The botanical names, plant parts, and voucher specimen numbers are listed in Table 1. Collection coordinates are provided in Table 4.

### 3.2. Preparation of the Extracts

Extracts were prepared within several hours after collecting the plants. Fresh plant material (5–110 g) was extracted with 100–200 mL of ethanol/ethyl acetate/water (1:1:1) at 60 °C for 1 h by using an ultrasound water bath apparatus. After vacuum filtration, ethanol and ethyl acetate were eliminated in a rotary vacuum evaporator at 60 °C. Finally, the remaining water solution was lyophilized to yield dried extracts. The extraction yield (%) for each extract (see identification number in Table 1) was: 1 (1.9%), 2 (3.0%), 3 (5.6%), 4 (4.5%), 5 (0.9%), 6 (2.9%), 7 (5.9%), 8 (4.0%), 9 (2.7%), 10 (5.5%), 11 (2.0%), 12 (2.9%), 13 (3.5%), 14 (8.5%), 15 (5.2%), 16 (7.4%), 17 (8.9%), 18 (Nd), 19 (6.7%), 20 (7.2%), 21 (2.4%), 22 (1.9%), 23 (2.4%), 24 (6.0%), 25 (14.8%), 26 (3.3%), 27 (1.0%), 28 (4.4%), 29 (6.8%), 30 (10.6%), 31 (4.9%), 32 (9.6%), 33 (4.6%), 34 (4.2%), 35 (4.0%), 36 (3.3%), 37 (4.2%), 38 (3.2%), 39 (5.9%), 40 (7.5%), 41 (5.3%), 42 (3.4%), 43 (2.7%), 44 (3.5%), 45 (2.4%), 46 (3.7%), 47 (2.6%), 48 (5.5%), 49 (0.9%), 50 (4.4%), 51 (5.5%), 52 (4.3%), 53 (5.8%), 54 (5.9%), 55 (Nd), 56 (8.4%), 57 (7.8%), 58 (5.5%), 59 (8.1%), 60 (6.5%), 61 (3.6%), 62 (10.7%), 63 (6.6%), 64 (9.0%), and 65 (9.9%). The extracts were stored in dark glass bottles and kept in a cool dark place. The first cytotoxicity experiment of the screening was carried out within the first month after preparing the extracts to avoid the possible degradation of active compounds. In the first cytotoxicity experiment, a stock solution of each extract was prepared in DMSO (100 mg/mL); a part of this solution was diluted in culture medium and immediately used to treat the cells. The stock solutions were aliquoted and frozen at −80 °C. The rest of independent cytotoxicity experiments were carried out using different aliquots to avoid freeze–thaw cycles.

### 3.3. Chemicals and Cell Lines

Cisplatin, MTT (3-(4,5-dimethylthiazol-2-yl)-2,5-diphenyltetrazolium bromide), and resazurin were bought from Sigma. MRC-5 (human fetal lung fibroblastic cells) and A549 (human non-small-cell lung cancer cells) were purchased from the European Collection of Cell Cultures. MDA-MB-231 (human triple-negative breast cancer cells) was purchased from the American Type Culture Collection (ATCC). UACC-62 (human BRAF mutant melanoma cells) was obtained from National Cancer Institute. HepG2 (human hepatocellular carcinoma cells), PC3 (human prostate cancer cells), HT29 (human colorectal cancer cells), MCF7 (human breast cancer cells), HeLa (human cervical carcinoma cells), NB4 (human acute promyelocytic leukemia cells), HL-60 (human acute promyelocytic leukemia cells), SW480 (human colon adenocarcinoma cells), and U2OS (human osteosarcoma cells) were kindly provided by Dr. Helleday (Karolinska Institute, Sweden). HNO97 (human tongue cancer cells), A64-CLS (human submaxillary gland adenoma cells), AN3Ca (human endometrial adenocarcinoma cells), Sk-OV-3 (human ovarian cancer cells), KATO III (human gastric cancer cells), Sk-Br-3 (HER2-positive breast cancer cells), T24 (human bladder cancer cells), Calu-1 (human squamous lung cancer cells), and MeWo (human BRAF wild-type melanoma cells) were purchased from Cell Lines Service (CLS). GAMG cells (human glioblastoma cells) were provided by Dr. A. Ayala (University of Seville). BJ-hTERT (hTERT-immortalized foreskin fibroblast BJ cells), BJ-SV40T (SV40T-transformed BJ-hTERT cells), and BJ RASV12 (H-RAS V12-transformed BJ-SV40T cells) were provided by Dr. Hahn (Dana-Farber Cancer Institute, USA) [14]. Cells were maintained in appropriate medium and propagated according to standard protocols. MRC-5, A549, MDA-MB-231, HepG2, HT29, MCF7, HeLa, SW48, U2OS, HNO97, A64-CLS, AN3Ca, SK-OV-3, Sk-Br-3, MeWo, GAMG, BJ-hTERT, BJ-SV40T, and BJ-RASV12, were maintained in Dulbecco’s modified Eagle’s medium (DMEM) high-glucose medium. PC3 and T24 were grown in DMEM-F12. UACC-62, NB4, HL60, and Calu-1 were maintained in RPMI 1640. KATO III were grown in Ham’s F12. All media were supplemented with 100 U/mL penicillin, 100 μg/mL streptomycin, and 10% fetal bovine serum. All cells were kept at 37 °C in a humidified atmosphere containing 5% CO_2_. Cell culture reagents were purchased from Biowest.

### 3.4. Cell Viability Assays

Exponentially growing cells (3000–5000 cells per well) were seeded in 96-well plates and were allowed to grow for 24 h. The cells were then exposed to several concentrations of the extracts or the positive controls cisplatin and 5-fluorouracil. After a treatment period of 72 h, cell viability was estimated with the MTT assay or the resazurin assay [2,15]. Due to the fact that plant extracts contain compounds (e.g., polyphenols) that may interfere with these assays, cell culture medium was removed before conducting these assays in all adherent cell lines.

The MTT assay is based on the ability of viable cells to convert the MTT compound (3-(4,5-dimethylthiazol-2-yl)-2,5-diphenyltetrazolium bromide) into an insoluble and purple formazan product. After an incubation period of the cells with the MTT and a solubilization step, the quantity of the colored product was measured with a plate reading spectrophotometer. Dead cells are metabolically inactive and cannot produce the colored product. Briefly, after the 72 h treatment period, the medium was removed, the cells were washed with PBS, and 125 μL MTT (1 mg/mL in medium) was added to each well. The plates were incubated for 4 h to allow viable cells to transform the MTT compound into an insoluble formazan product. This insoluble compound was solubilized by adding 80 μL 20% SDS in 0.02M HCl to each well and by incubating the plates overnight at 37 °C. Finally, optical densities were measured at 540 nm on a multi-well plate spectrophotometer reader (Multiskan EX Labsystems, Vantaa, Finland).

The resazurin assay is a redox-based colorimetric or fluorometric assay based on the capacity of viable cells to reduce the blue compound resazurin into the pink, fluorescent and soluble product resorufin. The quantity of resorufin produced is proportional to the number of viable cells. After treatment period, medium was removed and 150 μL of resazurin (20 µg/mL in medium) was added to each well for 5–6 h. The absorbance was measured at 540 nm and 620 nm on a multi-well plate spectrophotometer reader (Multiskan EX Labsystems, Vantaa, Finland).

In both assays, cell viability was calculated as a percentage in relation to untreated cells from at least two independent experiments. IC50 values were calculated by linear regression analysis after selecting the two concentrations leading to cell viability values immediately above and below 50%. With this approach, IC50 values always are within the experimentally observed concentration range [15,34,35,36,37]. After estimating cell viability and calculating IC50 values, results were expressed as mean ± standard error of the mean (SEM), and a t-test (paired, two tailed) was used for statistical analysis. A *p*-value > 0.05 was not considered statistically significant and was not represented by any symbol. A *p*-value ≤ 0.05 was considered statistically significant and was represented with an asterisk, two asterisks (*p* ≤ 0.01), or three asterisks (*p* ≤ 0.001). Since selectivity is the most relevant parameter to detect anticancer potential in vitro [3,4], selectivity indices were used to quantify this parameter. The selectivity index (S.I.) was calculated as the average of the IC50 value in the normal cell line (MRC-5) divided by the IC50 value in the cancer cell line (A549) obtained in each independent experiment [3].

## Figures and Tables

**Table 1:** Cytotoxic activity of plant extracts on lung cancer cells (A549) versus lung normal cells (MRC-5).

Extract	Plant Name (Family)	Part Used	Voucher Number (SEV)	Origin	IC50 (MTT) (Mean ± SEM, µg/mL)		S.I. (Mean ± SEM)
					A549 (Cancer)	MRC-5 (Normal)	
**1**	*Acis autumnalis* (L.) Sweet (Amaryllidaceae)	Whole plant	284654	Seville	32.9 ± 9.6	59.1 ± 18.0	1.9 ± 0.6
**2**	*Anagallis monelli* L. (Primulaceae)	Aerial Parts	284675	Cádiz	3.2 ± 1.4	0.6 ± 0.2	0.3 ± 0.2
**3**	*Anagallis monelli* L. (Primulaceae)	Root	284675	Cádiz	7.4 ± 5.2	0.9 ± 0.5	0.3 ± 0.3
**4**	*Anthyllis hamosa* Desf. (Leguminosae)	Whole plant	284685	Huelva	260.9 ± 14.7	317.7 ± 3.1	1.2 ± 0.1
**5**	*Aristolochia baetica* L. (Aristolochiaceae)	Fruits	284674	Seville	249.1 ± 2.5	649.9 ± 345.0	2.6 ± 1.4
**6**	*Aristolochia baetica* L. (Aristolochiaceae)	Leaves	284674	Seville	91.8 ± 70.5	66.0 ± 39.9	0.9 ± 0.3
**7**	*Armeria pungens* (Link) Hoffmanns. & Link (Plumbaginaceae)	Flowering aerial parts	284687	Huelva	143.4 ± 64.3	550.7 ± 352.2	3.6 ± 1.1
**8**	*Armeria velutina* Welw. ex Boiss. & Reut. (Plumbaginaceae)	Whole plant	284689	Huelva	25.7 ± 1.9	36.7 ± 0.9	1.4 ± 0.1
**9**	*Campanula lusitanica* L. (Campanulaceae)	Whole plant	284667	Seville	223.4 ± 51.2	263.7 ± 34.2	1.3 ± 0.4
**10**	*Cascabela thevetia* (L.) Lippold (Apocynaceae)	Leaves	284662	Seville	0.14 ± 0.02	1.6 ± 0.5	26.6 ± 11.6
**11**	*Centaurea sphaerocephala* L. (Compositae)	Whole plant	284683	Huelva	134.2 ± 82.4	160.4 ± 33.8	1.7 ± 0.8
**12**	*Centaurea sphaerocephala* L. (Compositae)	Flowers	284676	Cádiz	139.1 ± 40.1	108.7 ± 7.7	1.7 ± 1.1
**13**	*Centaurea sphaerocephala* L. (Compositae)	Leaves	284676	Cádiz	43.1 ± 11.1	114.7 ± 14.4	3.0 ± 0.8
**14**	*Cistus crispus* L. (Cistaceae)	Leaves	284660	Seville	52.4 ± 5.1	126.4 ± 34.3	2.5 ± 0.7
**15**	*Cistus crispus* L. (Cistaceae)	Root	284660	Seville	24.9 ± 0.7	58.8 ± 14.8	2.1 ± 0.7
**16**	*Cistus salviifolius* L. (Cistaceae)	Leaves	284653	Seville	56.6 ± 12.6	142.7 ± 20.4	3.0 ± 0.9
**17**	*Cleome violacea* L. (Cleomaceae)	Aerial Parts	284668	Seville	253.9 ± 39.9	261.1 ± 3.4	1.1 ± 0.2
**18**	*Digitalis purpurea* L. (Plantaginaceae)	Leaves	284691	Huelva	0.17 ± 0.15	1.06 ± 0.69	9.3 ± 0.5
**19**	*Dorycnium rectum* (L.) Ser. (Leguminosae)	Flowers	284690	Seville	102.1 ± 40.2	182.9 ± 46.1	4.3 ± 3.2
**20**	*Dorycnium rectum* (L.) Ser. (Leguminosae)	Leaves	284690	Seville	293.6 ± 21.2	322.1 ± 27.4	1.1 ± 0.2
**21**	*Echium gaditanum* Boiss. (Boraginaceae)	Aerial Parts	284684	Huelva	196.1 ± 107.2	325.6 ± 44.0	1.1 ± 0.1
**22**	*Elaeoselinum foetidum* (L.) Boiss. (Apiaceae)	Flowers	284670	Seville	119.0 ± 16.2	262.1 ± 8.7	2.3 ± 0.3
**23**	*Elaeoselinum foetidum* (L.) Boiss. (Apiaceae)	Leaves	284670	Seville	246.5 ± 25.5	295.0 ± 36.4	1.2 ± 0.0
**24**	*Erica arborea* L. (Ericaceae)	Bark	284657	Seville	32.2 ± 5.6	62.1 ± 28.9	1.8 ± 0.7
**25**	*Erica arborea* L. (Ericaceae)	Leaves	284657	Seville	45.7 ± 7.4	158.4 ± 43.6	3.9 ± 1.4
**26**	*Erophaca baetica* (L.) Boiss. (Leguminosae)	Leaves	284673	Seville	>1000	>1000	N.D.
**27**	*Frangula alnus* Mill. (Rhamnaceae)	Bark	284680	Huelva	32.8 ± 7.4	339.3 ± 74.1	12.4 ± 3.3
**28**	*Frangula alnus* Mill. (Rhamnaceae)	Leaves	284680	Huelva	28.6 ± 3.5	74.0 ± 38.5	2.4 ± 1.0
**29**	*Genista hirsuta* M.Vahl (Leguminosae)	Aereal Parts	284671	Seville	273.6 ± 5.8	363.9 ± 27.9	1.3 ± 0.1
**30**	*Halimium calycinum* (L.) K.Koch (Cistaceae)	Leaves	284656	Seville	47.4 ± 10.0	135.4 ± 23.6	4.3 ± 1.9
**31**	*Halimium calycinum* (L.) K.Koch (Cistaceae)	Root	284656	Seville	60.1 ± 17.7	101.1 ± 13.6	2.2 ± 0.8
**32**	*Halimium halimifolium* (L.) Willk. (Cistaceae)	Leaves	284659	Seville	47.9 ± 10.9	105.9 ± 21.4	2.7 ± 0.9
**33**	*Halimium halimifolium* (L.) Willk. (Cistaceae)	Root	284659	Seville	67.5 ± 16.0	110.2 ± 33.4	1.8 ± 0.8
**34**	*Hedysarum coronarium* L. (Leguminosae)	Flowers	284677	Cádiz	230.5 ± 54.6	306.9 ± 55.1	1.4 ± 0.1
**35**	*Hedysarum coronarium* L. (Leguminosae)	Fruits	284677	Cádiz	247.0 ± 3.2	294.5 ± 0.4	1.2 ± 0.0
**36**	*Hedysarum coronarium* L. (Leguminosae)	Leaves	284677	Cádiz	173.9 ± 60.6	262.0 ± 32.3	2.1 ± 0.9
**37**	*Iberis ciliata* subsp. *contracta* (Pers.) Moreno (Brassicaceae)	Whole plant	284688	Huelva	0.31 ± 0.06	2.31 ± 0.88	13.0 ± 4.7
**38**	*Jasione montana* L. (Campanulaceae)	Whole plant	284666	Seville	145.0 ± 26.0	301.6 ± 69.2	2.0 ± 0.1
**39**	*Juniperus macrocarpa* Sm. (Cupressaceae)	Monosperma cones	284682	Huelva	146.1 ± 125.9	>1000	>20
**40**	*Juniperus macrocarpa* Sm. (Cupressaceae)	Aerial Parts	284682	Huelva	3.7 ± 1.9	2.8 ± 1.0	0.8 ± 0.1
**41**	*Malcolmia lacera* (L.) DC. (Brassicaceae)	Whole plant	284664	Seville	322.3 ± 86.7	295.0 ± 5.9	1.0 ± 0.3
**42**	*Malva hispanica* L. (Malvaceae)	Aerial Parts	284669	Seville	>1000	703.1 ± 51.1	N.D.
**43**	*Ononis subspicata* Lag. (Leguminosae)	Whole plant	284695	Huelva	63.9 ± 23.6	232.3 ± 29.5	4.6 ± 1.4
**44**	*Ornithopus compressus* L. (Leguminosae)	Whole plant	284693	Seville	340.7 ± 28.4	583.8 ± 150.0	1.8 ± 0.6
**45**	*Ornithopus sativus* Brot. (Leguminosae)	Whole plant	284692	Seville	326.6 ± 37.5	699.0 ± 262.9	2.3 ± 1.1
**46**	*Pancratium maritimum* L. (Amaryllidaceae)	Bulb	284681	Huelva	3.4 ± 0.2	74.1 ± 56.1	19.7 ± 14.6
**47**	*Pycnocomon rutifolium* (Vahl) Hoffmanns. & Link (Caprifoliaceae)	Leaves	284678	Cádiz	71.4 ± 40.4	70.6 ± 18.1	2.2 ± 1.4
**48**	*Pycnocomon rutifolium* (Vahl) Hoffmanns. & Link (Caprifoliaceae)	Root	284678	Cádiz	262.5 ± 131,9	242.9 ± 56.7	1.1 ± 0.3
**49**	*Ranunculus peltatus* Schrank (Ranunculaceae)	Whole plant	284672	Seville	186.7 ± 54.6	170.1 ± 58.8	0.9 ± 0.1
**50**	*Rhamnus lycioides subsp. oleoides* (L.) Jahand. & Maire (Rhamnaceae)	Bark	284655	Seville	332.2 ± 61.9	599.4 ± 122.1	2.1 ± 0.8
**51**	*Rhamnus lycioides subsp. oleoides* (L.) Jahand. & Maire (Rhamnaceae)	Leaves	284655	Seville	151.5 ± 16.7	421.3 ± 176.5	3.1 ± 1.6
**52**	*Rhamnus lycioides subsp. oleoides* (L.) Jahand. & Maire (Rhamnaceae)	Root	284655	Seville	257.0 ± 76.6	442.3 ± 35.1	2.3 ± 0.8
**53**	*Scrophularia frutescens* L. (Scrophulariaceae)	Whole plant	284686	Huelva	281.5 ± 9.2	297.6 ± 8.6	1.1 ± 0.1
**54**	*Stauracanthus genistoides* (Brot.) G. Sampaio (Leguminosae)	Aerial Parts	284679	Huelva	278.4 ± 32.9	546.5 ± 53.5	2.0 ± 0.4
**55**	*Tamarix canariensis* Willd. (Tamaricaceae)	Flowers	284650	Seville	212.8 ± 10.2	336.3 ± 3.6	1.6 ± 0.1
**56**	*Tamarix canariensis* Willd. (Tamaricaceae)	Leaves	284650	Seville	92.7 ± 24.6	192.1 ± 33.0	2.2 ± 0.3
**57**	*Taxus baccata* L. (Taxaceae)	Leaves	284621	Seville	0.86 ± 0.27	146.9 ± 87.8	157.3 ± 110.6
**58**	*Tetraclinis articulata* (Vahl) Mast. (Cupressaceae)	Leaves	284663	Seville	0.37 ± 0.03	129.5 ± 64.0	378.3 ± 178.1
**59**	*Teucrium fruticans* L. (Lamiaceae)	Leaves	284658	Seville	157.1 ± 30.5	433.0 ± 112.3	2.8 ± 0.7
**60**	*Thymus mastichina* (L.) L. (Lamiaceae)	Whole plant	284694	Huelva	36.8 ± 7.7	277.7 ± 40.6	8.2 ± 2.0
**61**	*Tolpis barbata* (L.) Gaertn. (Compositae)	Whole plant	284665	Seville	86.5 ± 28.3	43.7 ± 12.2	0.5 ± 0.0
**62**	*Ulex parviflorus* Pourr. subsp. *parviflorus* (Leguminosae)	Flowers	284652	Seville	361.7 ± 89.6	876.1 ± 426.1	4.0 ± 2.9
**63**	*Ulex parviflorus* Pourr. subsp. *parviflorus* (Leguminosae)	Leaves	284652	Seville	99.6 ± 34.8	270.4 ± 85.3	3.4 ± 1.2
**64**	*Viburnum tinus* L. (Adoxaceae)	Fruits	284651	Seville	26.6 ± 6.5	65.4 ± 8.6	2.6 ± 0.3
**65**	*Viburnum tinus* L. (Adoxaceae)	Leaves	284651	Seville	234.8 ± 58.7	568.2 ± 73.0	2.6 ± 0.3
	Cisplatin (Standard anticancer drug)				10.5 ± 5.5 (µM)	25.4 ± 7.4 (µM)	4.2 ± 2.2
	5-Fluorouracil (Standard anticancer drug)				101.8 ± 7.7 (µM)	>1000 (µM)	>9.9

S.I.: selectivity index (calculated as the average of the IC50 value in the MRC-5 normal cell line divided by the IC50 value in the A549 cancer cell line obtained in each independent experiment); Nd: not determined.

**Table 2:** Cytotoxicity as IC50 values of selected extracts, cisplatin, and 5-FU on a panel of human cancer cell lines of different tissue origin and three genetically modified skin cell lines. Values are expressed as mean ± SEM.

	IC50 (Resazurin) (Mean ± SEM, µg/mL)						IC50 (Resazurin) (Mean ± SEM, µM)	
Cell Line	10	18	37	46	57	58	Cisplatin	5-FU
HepG2	24.6 ± 4.1	13.2 ± 3.0	19.4 ± 10.4	7.0 ± 1.3	632.6 ± 106.1	18.4 ± 17.1	3.5 ± 0.8	0.3 ± 0.2
SW480	20.7 ± 5.6	18.3 ± 1.2	1.2 ± 0.3	2.1 ± 0.4	2.2 ± 0.7	2.1 ± 0.8	2.0 ± 0.5	3.4 ± 1.0
U2OS	9.2 ± 1.5	8.8 ± 0.8	1.9 ± 0.2	2.8 ± 0.7	4.0 ± 1.2	2.1 ± 0.9	4.0 ± 1.0	7.2 ± 1.0
HeLa	4.7 ± 0.1	4.9 ± 0.2	3.5 ± 0.5	5.4 ± 0.4	2.5 ± 0.4	1.3 ± 0.6	4.8 ± 0.1	66.5 ± 27.0
PC3	4.7 ± 1.5	2.6 ± 0.4	5.6 ± 2.1	5.4 ± 1.2	123.8 ± 42.7	0.50 ± 0.04	7.3 ± 4.6	1.5 ± 0.8
MCF7	10.0 ± 0.5	6.5 ± 1.0	4.0 ± 0.9	5.9 ± 0.8	37.1 ± 4.9	0.7 ± 0.1	8.0 ± 3.0	1.1 ± 0.8
NB4	2.9 ± 0.7	1.8 ± 0.5	0.5 ± 0.2	1.2 ± 0.2	2.8 ± 0.2	0.2 ± 0.1	0.08 ± 0.04	2.7 ± 0.3
HL-60	3.5 ± 0.4	3.6 ± 0.3	0.8 ± 0.2	1.0 ± 0.3	3.3 ± 0.2	0.7 ± 0.3	0.2 ± 0.1	0.6 ± 0.5
BJ-hTERT	3.7 ± 0.4	2.8 ± 0.3	3.4 ± 0.5	3.4 ± 0.1	4.0 ± 0.6	0.8 ± 0.3	1.2 ± 0.4	2.6 ± 0.6
BJ-SV40T	2.9 ± 0.4	2.1 ± 0.4	2.9 ± 0.4	3.2 ± 0.4	2.8 ± 0.2	1.0 ± 0.4	0.7 ± 0.2	5.8 ± 0.7
BJ-RASV12	2.9 ± 0.4	1.8 ± 0.7	4.0 ± 1.4	2.0 ± 0.7	3.3 ± 0.2	0.6 ± 0.2	1.1 ± 0.1	1.7 ± 0.8

HepG2: human hepatocellular carcinoma; SW480: human colon adenocarcinoma; U2OS: human osteosarcoma; HeLa: human cervical carcinoma; PC3: human prostate adenocarcinoma; MCF7: human breast adenocarcinoma; NB4: human acute promyelocytic leukemia; HL-60: human acute promyelocytic leukemia; BJ-hTERT: hTERT-immortalized skin non-malignant BJ; BJ-SV40T: SV40T-transformed BJ-hTERT; BJ-RASV12: H-RAS V12-transformed BJ-SV40T. Extract numbers (**10**, **18**, **37**, **46**, **57**, and **58**) can be identified from Table 1.

**Table 3:** Cytotoxic activity of *Tetraclinis articulata* (Vahl) Mast. (**58**) against 14 cancer cell lines from a variety of tissues.

Cell Line	IC50 (Resazurin) (Media ± SEM, µg/mL)
	**58**
A64-CLS	4.7 ± 0.3
AN3Ca	4.5 ± 0.9
Calu-1	4.7 ± 0.2
GAMG	4.5 ± 0.6
HNO97	4.5 ± 0.3
HT29	4.4 ± 0.4
KATO III	4.4 ± 0.7
MDA-MB-231	4.7 ± 0.2
MeWo	4.3 ± 0.2
PC-3	4.5 ± 1.0
Sk-Br-3	4.9 ± 0.0
Sk-OV-3	4.7 ± 0.1
T24	4.5 ± 0.3
UACC-62	4.5 ± 0.2

A64-CLS: submaxillary gland adenoma; AN3Ca: endometrial adenocarcinoma; Calu-1: squamous lung cancer; GAMG: glioblastoma; HNO97: tongue cancer; HT29: colorectal cancer; KATO III: gastric cancer; MDA-MB-231: triple-negative breast cancer; MeWo: BRAF wild-type melanoma; PC-3: prostate cancer; Sk-Br-3: HER2-positive breast cancer; Sk-OV-3: ovarian cancer; UACC-62: BRAF mutant melanoma; and T24: bladder cancer.

**Table 4:** Collection coordinates of plants used in this work.

Extract	Plant Name	Collection Coordinates
**1**	*Acis autumnalis* (L.) Sweet	37°14′22.06″ N 6°11′37.85″ W
**2–3**	*Anagallis monelli* L.	36°36′15.24″ N 6°16′2.76″ W
**4**	*Anthyllis hamosa* Desf.	37°04′25.2″ N 6°41′19.68″ W
**5–6**	*Aristolochia baetica* L.	37°14′16.68″ N 6°11′48.38″ W
**7**	*Armeria pungens* (Link) Hoffmanns. & Link	37°04′13.73″ N 6°41′16.97″ W
**8**	*Armeria velutina* Welw. ex Boiss. & Reut.	37°02′33.33″ N 6°35′53.85″ W
**9**	*Campanula lusitanica* L.	37°14′14.46″ N 6°11′55.8″ W
**10**	*Cascabela thevetia* (L.) Lippold	37°22′59.8″ N 5°59′27.36″ W
**11–13**	*Centaurea sphaerocephala* L.	37°05′40.18″ N 6°43′37.9″ W
**14–15**	*Cistus crispus* L.	37°14′22.06″ N 6°11′37.85″ W
**16**	*Cistus salvifolius* L.	37°14′19.74″ N 6°11′40.71″ W
**17**	*Cleome violacea* L.	37°14′23.37″ N 6°11′52.72″ W
**18**	*Digitalis purpurea* L.	37°27′30.78″ N 6°41′20.3″ W
**19–20**	*Dorycnium rectum* (L.) Ser.	37°20′14.71″ N 5°51′27.13″ W
**21**	*Echium gaditanum* Boiss.	37°04′11.69″ N 6°41′17.16″ W
**22–23**	*Elaeoselinum foetidum* (L.) Boiss.	37°14′15.72″ N 6°11′50.86″ W
**24–25**	*Erica arborea* L.	37°14′38.35″ N 6°11′49.92″ W
**26**	*Erophaca baetica* (L.) Boiss.	37°14′17.72″ N 6°11′46.41″ W
**27–28**	*Frangula alnus* Mill.	37°05′40.46″ N 6°43′34.47″ W
**29**	*Genista hirsuta* M. Vahl	37°14′16.70 “N 6°11′49.05″ W
**30–31**	*Halimium calycinum* (L.) K. Koch	37°14′21.27N 6°11′38.12″ W
**32–33**	*Halimium halimifolium* (L.) Willk.	37°14′23.43″ N 6°11′38.77″ W
**34–36**	*Hedysarum coronarium* L.	36°36′39.92″ N 6°16′46.6″ W
**37**	*Iberis ciliata* subsp. *contracta* (Pers.) Moreno	37°04′47.25″ N 6°41’13.82″ W
**38**	*Jasione montana* L.	37°13′45.76″ N 6°9′16.08″ W
**39–40**	*Juniperus macrocarpa* Sm.	37°04′13.53″ N 6°41′16.34″ W
**41**	*Malcolmia lacera* (L.) DC.	37°13′45.76″ N 6°9′16.08″ W
**42**	*Malva hispanica* L.	37°14′16.09″ N 6°11′51.89″ W
**43**	*Ononis subspicata* Lag.	37°04′45.90″ N 6°41′14.14″ W
**44**	*Ornithopus compressus* L.	37°13′45.76″ N 6°9′16.08″ W
**45**	*Ornithopus sativus* Brot.	37°13′45.76″ N 6°9′16.08″ W
**46**	*Pancratium maritimum* L.	37°04′11.14″ N 6°41′16.29″ W
**47–48**	*Pycnocomon rutifolium* (Vahl) Hoffmanns. & Link	36°36′12.69″ N 6°15′54.45″ W
**49**	*Ranunculus peltatus* Schrank	37°13′45.76″ N 6°9′16.08″ W
**50–52**	*Rhamnus lycioides* subsp. *oleoides* (L.) Jahand. & Maire	37°14′25.18″ N 6°11′38.35″ W
**53**	*Scrophularia frutescens* L.	37°04′47.25″ N 6°41′13.82″ W
**54**	*Stauracanthus genistoides* (Brot.) G. Sampaio	37°04′44.55″ N 6°41′16.05″ W
**55–56**	*Tamarix canariensis* Willd.	37°15′45.64″ N 5°59′50.9″ W
**57**	*Taxus baccata* L.	37°22′27″ N 5°59′19″ W
**58**	*Tetraclinis articulata* (Vahl) Mast.	37°22′22.18″ N 5°59′10.75″ W
**59**	*Teucrium fruticans* L.	37°14′42.60″ N 6°11′52.78″ W
**60**	*Thymus mastichina* (L.) L.	37°02′33.33″ N 6°35′53.85″ W
**61**	*Tolpis barbata* (L.) Gaertn.	37°13′45.76″ N 6°9′16.08″ W
**62–63**	*Ulex parviflorus* Pourr. subsp. *parviflorus*	37°13′48.06″ N 6°1′31.4″ W
**64–65**	*Viburnum tinus* L.	37°22′27″ N 5°59′19″ W

## Data Availability

Not applicable.

## Supplementary Materials

The following are available online at https://www.mdpi.com/article/10.3390/plants10102193/s1. Figure S1: Comparison of cytotoxicity of *Tetraclinis articulata (Vahl) Mast*. extract (**58**) assessed by MTT and resazurin assays. A549 and MRC-5 cells were treated for 72 h. Figure S2: *Tetraclinis articulata (Vahl) Mast*. (Cupressaceae). General view of the plant (a) and the plant material (**b**) used in the present study.
