# Supplementary figures and images for: Screening for Selective Anticancer Activity of 65 Extracts of Plants Collected in Western Andalusia, Spain
Source: Plants (Basel). 2021 Oct 15;10(10):2193. doi: 10.3390/plants10102193 (PMC8537044; doi:10.3390/plants10102193)

58

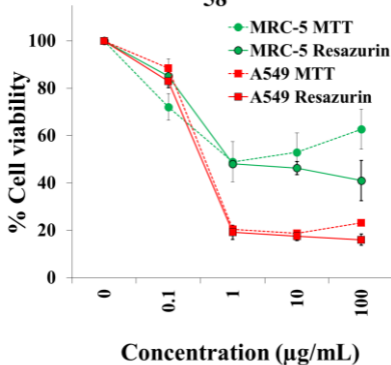

Supplement: Supplementary file 1 [file plants-10-02193-s001.zip › Figure S1.pdf]

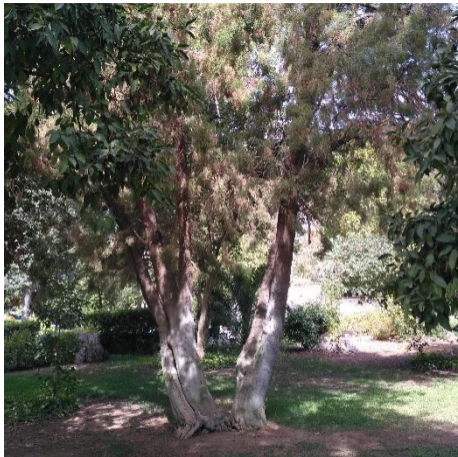

(a)

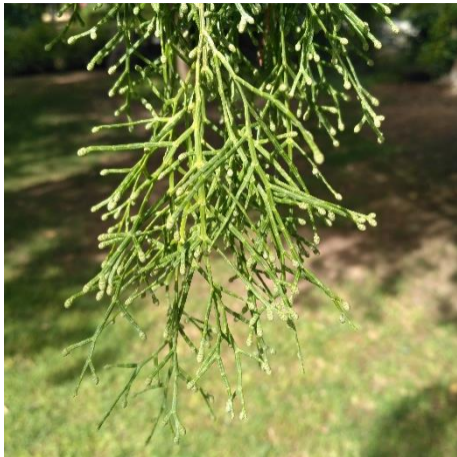

(b)

Supplement: Supplementary file 1 [file plants-10-02193-s001.zip › Figure S2.pdf]
